# Potential of NRF2 Pathway in Preventing Developmental and Reproductive Toxicity of Fine Particles

**DOI:** 10.3389/ftox.2021.710225

**Published:** 2021-09-13

**Authors:** Ying-Ji Li, Ken Takeda, Masayuki Yamamoto, Tomoyuki Kawada

**Affiliations:** ^1^ Department of Hygiene and Public Health, Nippon Medical School, Tokyo, Japan; ^2^ Faculty of Pharmaceutical Sciences, Sanyo-onoda City University, Sanyo-Onoda, Japan; ^3^ Medical Biochemistry, Tohoku University Graduate School of Medicine, Sendai, Japan

**Keywords:** ultrafine and nano-sized particles, oxidative stress, antioxidants, fetal development, *NRF2* SNP

## Abstract

Air pollution is associated with significant adverse health effects. Recent studies support the idea that inhalation of fine particles can instigate extrapulmonary effects on the cardiovascular system through several pathways. The systemic transfer of ultrafine particles (UFPs) or soluble particle components (organic compounds and metals) is of particular concern. An integral role of reactive oxygen species (ROS)-dependent pathways has been suggested in systemic inflammatory responses and vascular dysfunction at the molecular level. Accumulating lines of evidence suggest that fine particles affect fetal development, giving rise to low birth weight and a reduction in fetal growth, and also affect the immune, cardiovascular, and central nervous systems. Oxidative stress plays an important role in fine particles toxicity; pre-treatment with antioxidants partially suppresses the developmental toxicity of fine particles. On the other hand, Nuclear factor erythroid-derived 2-like 2 (Nfe2l2), also known as NRF2, is a transcription factor essential for inducible and/or constitutive expression of phase II and antioxidant enzymes. Studies using *Nrf2*-knockout mice revealed that NRF2 dysfunction is intimately involved in the pathogenesis of various human diseases. Multiple single nucleotide polymorphisms (SNPs) have been detected in human *NRF2* locus. An *NRF2* gene SNP (−617C > A; rs6721961), located in the upstream promoter region, affects the transcriptional level of NRF2 and thereby the protein level and downstream gene expression. It has been reported that the SNP-617 is associated with various diseases. The onset and exacerbation of the diseases are regulated by genetic predisposition and environmental factors; some people live in the air-polluted environment but are not affected and remain healthy, suggesting the presence of individual differences in the susceptibility to air pollutants. NRF2 polymorphisms may also be associated with the fetal effects of fine particles exposure. Screening high-risk pregnant women genetically susceptible to oxidative stress and prevention by antioxidant interventions to protect fetal development in air-polluted areas should be considered. This article reviews the recent advances in our understanding of the fetal health effects of fine particles and describes potential chemoprevention *via* the NRF2 pathway to prevent the developmental and reproductive toxicity of fine particles.

## Introduction

The strongest evidence from many epidemiological studies linking air pollution to human health centers on particulate components (Dockery et al., 1993; Gehring et al., 2010; Krishnan et al., 2012; Xia et al., 2013). Fine particles are classified according to their aerodynamic diameter into size fractions such as particulate matter 2.5 (PM_2.5_, diameter fine inhalable particles, with diameters that are generally 2.5 micrometers and smaller) and ultrafine (nano-sized) particles (UFPs, fine inhalable particles, with diameters that are generally 0.1 micrometers and smaller) (Araujo and Nel, 2009). Recent studies support the hypothesis that inhalation of fine particles can instigate extrapulmonary effects on the cardiovascular system through several pathways. Many epidemiological studies have attempted to clarify the association between PM_2.5_ and extrapulmonary disorders such as ischemic cardiovascular diseases (Lipsett et al., 2011; Hayes et al., 2020), arteriosclerosis (Hoffmann et al., 2007; Kaufman et al., 2016), neurological disorders (Zanobetti et al., 2014; Grande et al., 2020), diabetes (Raaschou-Nielsen et al., 2013; Balti et al., 2014), fetal development, and reproduction (Volk et al., 2013; DeFranco et al., 2016) (Table 1). PM_2.5_ in the atmosphere contain a lot of UFPs. Systemic transfer of UFPs or soluble particle components (organic compounds and metals) is of most concern in this context. An integral role of reactive oxygen species (ROS)-dependent pathways has been suggested in systemic pro-inflammatory responses and vascular dysfunction at the molecular level (Brook et al., 2010). Although epidemiological studies on the health effects of UFPs also are needed, exposure assessment for atmospheric UFPs is complex (Sioutas et al., 2005) and emerging evidence on UFPs health effects has mainly led by experimental studies using animals (Sugamata et al., 2006; Shimizu et al., 2009; Takeda et al., 2009; Takahashi et al., 2010; Kubo-Irie et al., 2014; Onoda et al., 2014; Shimizu et al., 2014; El-Sayed et al., 2015; Mitsunaga et al., 2016; Onoda et al., 2017) and cells (Xia et al., 2004; Mo et al., 2009; Li et al., 2013).

**TABLE 1:** Epidemiological studies linking PM_2.5_ exposure with extrapulmonary disorders.

Disorders	Major findings	References
Ischemic cardiovascular	Increased risks of incident stroke as well as ischemic heart disease mortality	Lipsett et al. (2011)
	Associated with the risks of ischaemic heart disease and stroke mortality	Hayes et al. (2020)
Arteriosclerosis	Associated with the degree of coronary atherosclerosis	Hoffmann et al. (2007)
	Associated with progression in coronary calcification, consistent with acceleration of atherosclerosis	Kaufman et al. (2016)
Neurological disorders	Associated with a higher risk of dementia	Grande et al. (2020)
	Increased the risk of hospitalizations for Parkinson’s disease and diabetes, and of all-cause mortality	Zanobetti et al. (2014)
Diabetes	Increased risk for type 2 diabetes	Balti et al. (2014)
	Associated with mortality from diabetes	Raaschou-Nielsen et al. (2013)
Fetal development and reproduction	Associated with autism	Volk et al. (2013)
	Increased risk of preterm birth	DeFranco et al. (2016)

On the other hand, Nuclear factor erythroid-derived 2-like 2 (Nfe2l2), also known as NRF2, is a transcription factor essential for the inducible and/or constitutive expression of phase II and antioxidant enzymes (Itoh et al., 1997). Recent studies using *Nrf2*-knockout mice revealed that NRF2 dysfunction is intimately involved in the pathogenesis of various human diseases after exposure to diesel exhaust (DE), and *Nrf2* knockout mice are highly sensitive to oxidative stress caused by DE (Li et al., 2008; Li et al., 2010; Li et al., 2017; Li et al., 2020). Most DE particles (DEPs) are contained in fine particulates and contain nano-sized carbon particles at their core (Araujo and Nel, 2009). In this regard, it is interesting to note that multiple single nucleotide polymorphisms (SNPs) have been detected in human *NRF2* locus. An *NRF2* gene SNP (−617C > A; rs6721961), located in the upstream promoter region, affects the transcriptional level of NRF2 and thereby the protein level and downstream gene expression (Yamamoto et al., 2004). It has been reported that the SNP-617 is associated with various diseases (Marzec et al., 2007; von Otter et al., 2010; Ungvári et al., 2012; Suzuki et al., 2013; Okano et al., 2013; Shimoyama et al., 2014; Wang et al., 2015). It is speculated that the reduction of protein levels and downstream gene expression by the *NRF2* gene SNP may increase susceptibility to oxidative stress caused by fine particulates exposure.

Many studies have suggested that fine particulates air pollution is related to developmental and reproductive (Gilboa et al., 2005; Shimizu et al., 2009; Takeda et al., 2009; Takahashi et al., 2010; Dadvand et al., 2013; Volk et al., 2013; Yokota et al., 2013; Onoda et al., 2014; Kubo-Irie et al., 2014; Symanski et al., 2014; El-Sayed et al., 2015; Tachibana et al., 2015; DeFranco et al., 2016; Mitsunaga et al., 2016; Yokota et al., 2016; Carré et al., 2017; Chen et al., 2017; Martens et al., 2017; Onoda et al., 2017). The impact of air pollutants on the next generation is of great concern, and preventive measures are required. This article reviews the recent advances in our understanding of the fetal health effects of fine particulates and discusses the potential chemoprevention *via* the NRF2 pathway to prevent the developmental and reproductive toxicity of fine particulates.

## Characteristics of Fine Particulates

Particulate matter is a complex mixture of organic and inorganic chemicals, including metals and particulates (Mo et al., 2009), and is composed of heterogeneous compounds of different sizes, chemical compositions, surface areas, concentrations, and sources. Fine particles are classified according to their aerodynamic diameter into size fractions such as PM_2.5_ (particulate matter of diameter <2.5 μm) and UFPs (particulate matter of diameter <0.1 μm). These particles are derived from various sources and by various mechanisms as shown in Table 2 (Araujo and Nel, 2009). These are just physical definitions, and the chemical components and the subsequent toxic characteristics of fine particles vary by country or region. Differences in energy structure, economic development, climate classification, etc., determine the type of air pollution and the chemical composition of fine particles (Pan et al., 2014). At the cellular level, fine particles various mechanisms involve free radical production (by transition metals and organic compounds), oxidative stress, cytokine release, inflammation, etc., (Araujo and Nel, 2009).

**TABLE 2:** Classification of fine particles based on size *.

Particle	Aerodynamic	Sources	Mode of generation
Fine particles (PM2.5)	<2.5	Power plants, oil refineries, wildfires, residential fuel combustion, tailpipe and brake emissions	Gas-to-particle conversion by condensation, coagulation (accumulation mode)
Ultrafine particles (UFPs)	<0.1	Fuel combustion (diesel, gasoline) and tailpipe emissions from mobile sources (motor vehicles, aircrafts, ships)	Fresh emissions, secondary photochemical reactions (nucleation mode)

* Cited from Araujo JA and Nel, 2009.

Several studies have shown that UFPs are more toxic than larger particles (Li et al., 2003; Donaldson et al., 2005). UFPs are important because when compared with larger particles, they have order of higher particle number concentration and surface area, and larger concentrations of adsorbed or condensed toxic air pollutants (oxidant gases, organic compounds, transition metals) per unit mass (Sioutas et al., 2005). Also facilitates their intake during breathing, and their mass ratio greatly enhances their chemical/catalytic reactivity compared to large-sized particles (Mikami et al., 2014). UFPs are not as easily phagocytized by alveolar macrophages as larger particles. They may enter the blood circulation, and translocation to extra-pulmonary tissues (Frampton, 2001; Sioutas et al., 2005; Brook et al., 2010). UFPs, with their high surface area, can carry large amounts of adsorbed or condensed toxic air pollutants, such as oxidant gas, organic compounds and transition metals (Oberdörster and Utell, 2002). It is also reported that the induction of mitochondrial dysfunction caused by DEPs and UFPs are mediated by adsorbed chemicals quinones and aromatics rather than the particles themselves (Xia et al., 2004).

DE emissions, are a major source of UFPs in urban environments, and it is these particles that have the capacity to induce the most significant health effects (Wåhlin et al., 2001; Miller and Newby, 2020). Previous studies have shown that DE exposure can have many adverse effects on the cardiovascular system, both acutely and chronically (Miller and Newby, 2020). DEPs have a complex structure characterized by nano-sized carbon particles at their core with adsorbed organic compounds such as polyaromatic hydrocarbons (PAHs) and quinones. The PAHs and their oxygenated derivatives (e.g., quinones) have attracted attention because they are able to participate in the redox cycle and generate ROS in target cells (Takizawa, 2004). Therefore, DEPs have been extensively used in studies as a model air pollutant. However, it has been also reported that standardized DEPs such as standard reference material (SRM)-2,975 are not suitable to represent traffic emissions and typical ambient particulate matter used in toxicological studies (Farahani et al., 2021). Since the actual composition of air pollutants depends on the region, the DEPs used in the research has a limit as a model of air pollutants.

## Fine Particles Affect Fetal Health

Studies support an association for fine particles and fetal health (Table 3). Exposure to PM_2.5_ affects development and reproduction as have been documented in epidemiological reports (Dadvand et al., 2013; Volk et al., 2013; Symanski et al., 2014; DeFranco et al., 2016; Martens et al., 2017) and supported by data from animal-model experiments (Yokota et al., 2013; Tachibana et al., 2015; Yokota et al., 2016; Chen et al., 2017). Epidemiological reports revealed that exposure to fine particulates during pregnancy is associated with autism (Volk et al., 2013) and causes changes in reproductive function (Carré et al., 2017). Volk, et al. (2013) Reported that exposure to traffic-related air pollution, such as PM_2.5_, during pregnancy and the first year of life was associated with autism. This study is based on a case-control study and includes data from 279 children with autism and 245 children with typical development in California (Volk et al., 2013). Exposure to fine particulates during pregnancy is also associated with biological aging (Martens et al., 2017), preterm birth (Symanski et al., 2014; DeFranco et al., 2016), and low birth weight (Dadvand et al., 2013). Similarly, animal-model experiments suggested that exposure to DE during pregnancy alters energy metabolism (Chen et al., 2017) and nervous function (Tachibana et al., 2015; Yokota et al., 2016). In animal model experiments, exposure to UFPs smaller than 0.1 µm diameter appeared critical for the development and reproduction, such as changes in reproductive function (Takeda et al., 2009; Kubo-Irie et al., 2014) and immune responses (Shimizu et al., 2014; El-Sayed et al., 2015). Sugamata, et al. (2006) reported that maternal exposure to DE which contain nano-sized carbon particles at their core, alters the ultrastructure of perivascular macrophages (PVMs) and surrounding tissues in the brain of mouse offspring. Onoda et al. (2014) found that maternal exposure to ultrafine carbon black altered the phenotype of PVMs and astrocytes close to blood vessels in offspring mice. This results suggest that maternal ultrafine carbon black exposure may associated with increased risk of dysfunction in the offspring brain (Onoda et al., 2014). Umezawa et al. (2014) also reported that the degree of the risk on offspring depends on the type of nanoparticles. Furthermore, many other animal studies also reported that exposure to UFPs provokes fetal brain dysfunction (Shimizu et al., 2009; Takeda et al., 2009; Takahashi et al., 2010; Mitsunaga et al., 2016; Onoda et al., 2017). Ambient fine particles contain a large proportion of UFPs; due to their small size, UFPs have high physicochemical reactivity and show unique behaviors *in vivo* including the following three potential pathways: 1) release of proinflammatory mediators from lung cells, 2) affect autonomic nervous system balance by particle interactions with lung receptors or nerves, and 3) translocation of UFPs and soluble particle components into the systemic circulation. Therefore, inhaled UFPs can reach the alveolar region and extrapulmonary organs (Brook et al., 2010). It is also possible that the UPFs contained in the fine particles may affect the foetation. UFPs also have higher permeability than large-sized particles; this facilitates the translocation of particles from mother to infant (Mikami et al., 2014).

**TABLE 3:** Studies linking fine particulates exposure with fetal health.

Study	Fine particulates	Major findings	References
Epidemiological study	Atmospheric PM_2.5_	Associated with autism	Volk et al. (2013)
		Associated with preterm birth	DeFranco et al. (2016)
			Symanski et al. (2014)
		Associated with shorter telomere length	Martens et al. (2017)
		Associated with low birth weight	Dadvand et al. (2013)
Animal study	DEPs	Provokes fetal brain dysfunction	Tachibana et al. (2015)
			Yokota et al. (2016)
			Yokota et al. (2013)
			Sugamata et al. (2006)
		Alters energy metabolism	Chen et al. (2017)
	UFPs	Provokes fetal brain dysfunction	Onoda et al. (2017)
			Onoda et al. (2014)
		Damage the genital and cranial nerve systems	Takeda et al. (2009)
			Kubo-Irie et al. (2014)
		Changes immune responses	El-Sayed et al. (2015)
			Shimizu et al., (2014)

## Mechanisms of Fine Particles Biological Activity: Role of Oxidative Stresses Induced by Fine Particles

There is increasing evidence that fine particulate pollutants induce inflammatory responses, and these proinflammatory effects have been linked to the ability of fine particulate, such as DEPs, to generate ROS and oxidative stress in bronchial epithelial cells (Takizawa et al., 1999; Hashimoto et al., 2000), macrophages (Li et al., 2004). Li et al. (2003) demonstrated that the increased biological potency of UFPs is related to the content of redox cycling organic chemicals and their ability to damage mitochondria. UFPs were collected by ambient particle concentrators in the Los Angeles basin in California and used to study their chemical composition in parallel with assays for generation of ROS and ability to induce oxidative stress in macrophages and epithelial cells. UFPs exposure induces oxidative stress by promoting cellular heme oxygenase-1 (HO-1) expression; HO-1 depletes intracellular glutathione and is a sensitive marker of oxidative stress. The results showed that HO-1 expression directly correlated with the high organic carbon and polycyclic aromatic hydrocarbon (PAH) content of UFPs; PAHs have been identified in placental tissue and umbilical cord blood from neonates. Detection of damaged DNA in cord blood indicates that exposure to these pollutants in the environment can cause fetal damage (RavindraMittal and Van Grieken, 2001). Oxidative stress likely plays an important role in nanoparticle toxicity, as pre-treatment with antioxidants partially suppresses the developmental toxicity of nanoparticles (Wang et al., 2010). PAHs, usually bound to fine particles and UFPs, increase the risk of intrauterine growth retardation. The permeability and increased ROS generation (which induces oxidative stress in cells) of small particles are the mechanisms underlying these harmful effects (Wåhlin et al., 2001).

## NRF2 as a Key Trascription Factor Preventing Cellular Damage in Response to Oxidative Stress

**FIGURE 1:**
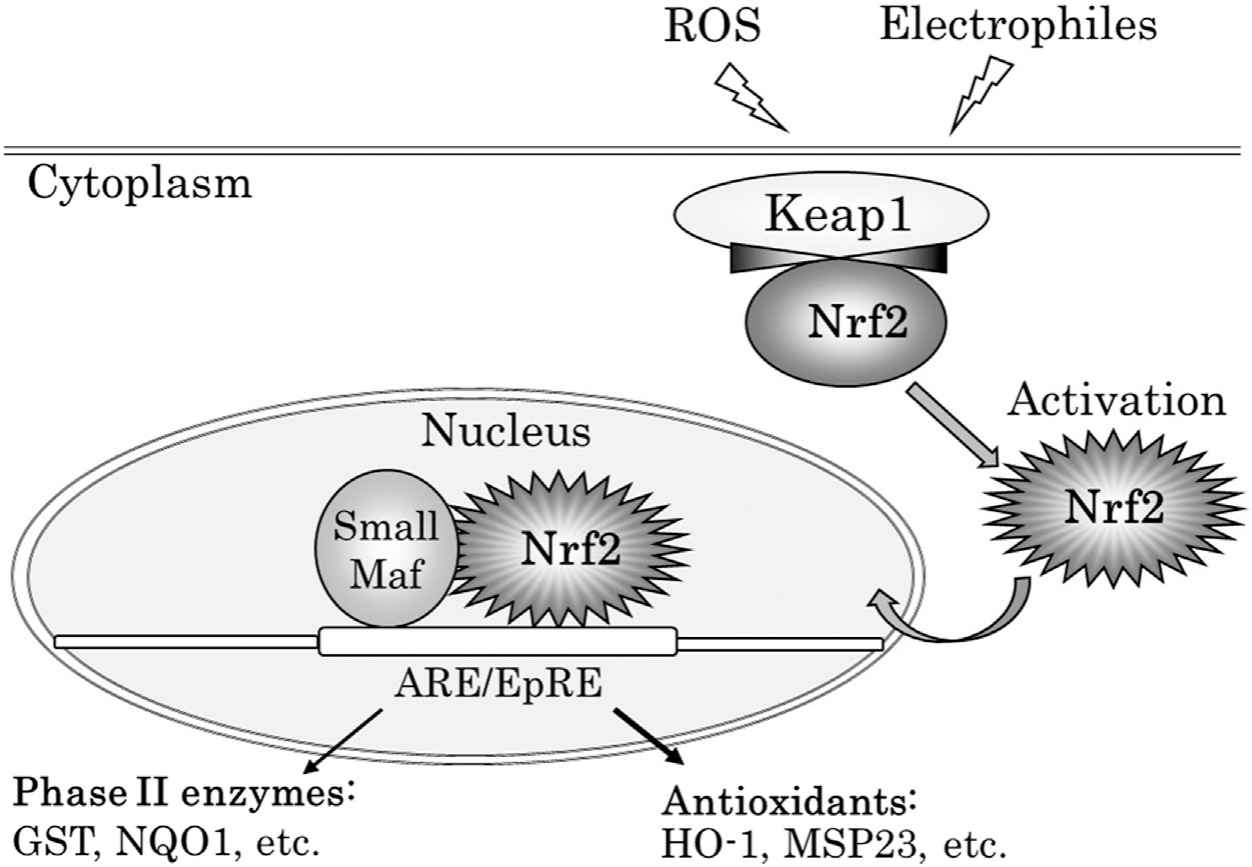
Activation of Nrf2 with ROS or electrophiles, and expression of Phase II enzyme genes and antioxidant stress protein genes *via* ARE/EpRE. Under non-stressed conditions, the transcription factor Nrf2 is constitutively degraded by binding to Keap1. ROS or electrophilic attack leads to the dissociation of Nrf2 from Keap1. Activated Nrf2 protein is then translocated into the nucleus and many genes encoding detoxifying and antioxidant enzymes are expressed. ROS: reactive oxygen species, ARE: antioxidant response element, EpRE: electrophile responsive element, GST: glutathione S-transferase, NQO1: NAD(P)H quinone dehydrogenase 1, HO-1: heme oxygenase-1, MSP23: macrophage 23-kDa stress protein.

Nrf2 is a key transcription factor that is essential for the induction and/or constitutive expression of phase II enzymes and antioxidants in response to ROS or electrophile (Itoh et al., 1997). Nrf2 possesses a Neh2 domain, which is recognized by Keap1 (Kelch-like ECH-associated protein 1) and acts as a degron–an amino acid sequence, which signals degradation (Itoh et al., 1999) Upon exposure to oxidative and electrophilic stress, Nrf2 is activated, and accumulates in nuclei, forms a heterodimer with a member of small Maf proteins, and binds to antioxidant ⁄ electrophile responsive elements (ARE⁄EpRE) located in its target genes (Rushmore et al., 1991; Prestera et al., 1995). This leads to the induction of a battery antioxidants (Ishii et al., 1993) and phase II detoxifying enzyme genes (Itoh et al., 1997) (Figure 1). Cytoprotective pathways are induced by the Nrf2 transcription signal pathway at the lowest levels of oxidative stress from DEPs and can induce the transcription of antioxidant genes in the earliest level of defense. Nrf2 regulates antioxidant defense that is constituted as the main defense action against the pro-inflammatory and oxidizing effects of DEPs (Li et al., 2004). Xiao et al. (2003) showed the hierarchical oxidative stress model in response to redox cycling DEPs components *in vitro*: Cytoprotective pathways are induced by the Nrf2 transcription signal pathway at the lowest levels of oxidative stress, and this may constitute the first tier of a hierarchical oxidative stress response, as is in the production of heme oxygenase (HO)-1. If these enzymes fail to neutralize the effects of ROS, proinflammatory effects constitute a second tier or superimposed level of oxidative stress. The final tier or superimposed level of oxidative stress is cytotoxicity, including the initiation of programmed cell death. (Xiao et al., 2003). DNA adduct formation is accelerated in the lungs of *Nrf2* knockout mice exposed to DE inhalation (Aoki et al., 2001). Previous studies suggest that oxidative stress induced by DE inhalation is associated with airway inflammation (Li et al., 2008), allergic asthma (Li et al., 2010), pulmonary fibrosis (Li et al., 2017), and airway innate immune responses (Li et al., 2020) evidenced in experiments using *Nrf2* knockout mice. Nrf2 also played an important role in mediating the adjuvant effect of UFPs at the level of functional dendritic cells (Li et al., 2013).

## NRF2 Polymorphism

Multiple single nucleotide polymorphisms (SNPs) have been identified in human *NRF2* (Yamamoto et al., 2004). The *NRF2* gene SNP (-617C > A; rs6721961) located in the upstream promoter region affects the transcriptional level of *NRF2* and thus the protein level and downstream gene expression. Suzuki et al. (2013) reported that minor A/A homozygotes of *NRF2* rSNP-617 exhibit significantly decreased *NRF2* gene expression. SNP-617 was found to be associated with a higher risk of oxidant-induced acute lung injury in humans (Yamamoto et al., 2004; Marzec et al., 2007). Individuals with *NRF2* polymorphisms have been reported to be at greater risk of developing acute lung injury (Marzec et al., 2007), Parkinson’s disease (von Otter et al., 2010), diabetes mellitus (Wang et al., 2015), chronic obstructive pulmonary disease (Hua et al., 2010), breast cancer (Hartikainen et al., 2012), cerebrovascular disease (Kunnas et al., 2016), and vascular stiffness (Shimizu et al., 2020). The presence of *NRF2* polymorphisms correlates significantly with the incidence of non-small cell lung cancer, especially in smokers (Suzuki et al., 2013), and is also related to air pollution and childhood asthma (Ungvári et al., 2012) (Table 4). Thus, personalized medicine based on *NRF2* polymorphisms might be effective to treat environmental pollutant-induced diseases.

**TABLE 4:** Representative diseases associated with *NRF2* polymorphism.

Diseases	References
Non-small cell lung cancer	Suzuki et al. (2013)
Acute lung injury	Marzec et al. (2007)
Air pollution and childhood asthm	Ungvári et al. (2012)
Parkinson’s disease	von Otter et al. (2010)
Type 2 diabetes	Wang et al. (2015)
Chronic obstructive pulmonary disease	Hua et al., (2010)
Breast cancer	Hartikainen et al. (2012)
Cerebrovascular disease	Kunnas et al. (2016)
Vascular stiffness	Shimizu et al. (2020)

As mentioned above, the NRF2 transcription factor controls cellular adaptation/protection to ROS and electrophiles by inducing antioxidant and detoxification genes. Under non-stressed conditions, the transcription factor Nrf2 is constitutively degraded by binding to Keap1 (Itoh et al., 1997; Itoh et al., 1999; Kobayashi et al., 2004). Oxidative stress and/or electrophilic attack leads to the dissociation of Nrf2 from Keap1 (Kobayashi et al., 2006); the Nrf2 protein is then translocated into the nucleus (Iso et al., 2016), and many genes encoding detoxifying and antioxidant enzymes are regulated by Nrf2 (Motohashi and Yamamoto, 2004; Kobayashi and Yamamoto, 2006; Suzuki et al., 2020). Notably, changes in *Nrf2* transcript level alter the Nrf2 protein level, even in the basal state, in which Keap1 actively degrades Nrf2. When the appropriate *Nrf2* transcript is supplied, the Nrf2 protein is maintained at low levels by Keap1-mediated degradation under basal conditions, and constant levels of Nrf2 protein are accumulated after inactivation by Keap1. When *Nrf2* transcription levels are low, Nrf2 protein is reduced in both basal and stress-induced states. When *Nrf2* transcription levels are high, large amounts of Nrf2 are produced in both basal and stress-induced states (Suzuki et al., 2013). This study suggests that the transcription level of the *NRF2* gene is indeed important for the roles that NRF2 plays in cytoprotection (Okano et al., 2013).

### A Personalized Prevention of Fine Particles-Induced Oxidative Stress Based on NRF2 Polymorphism

**FIGURE 2:**
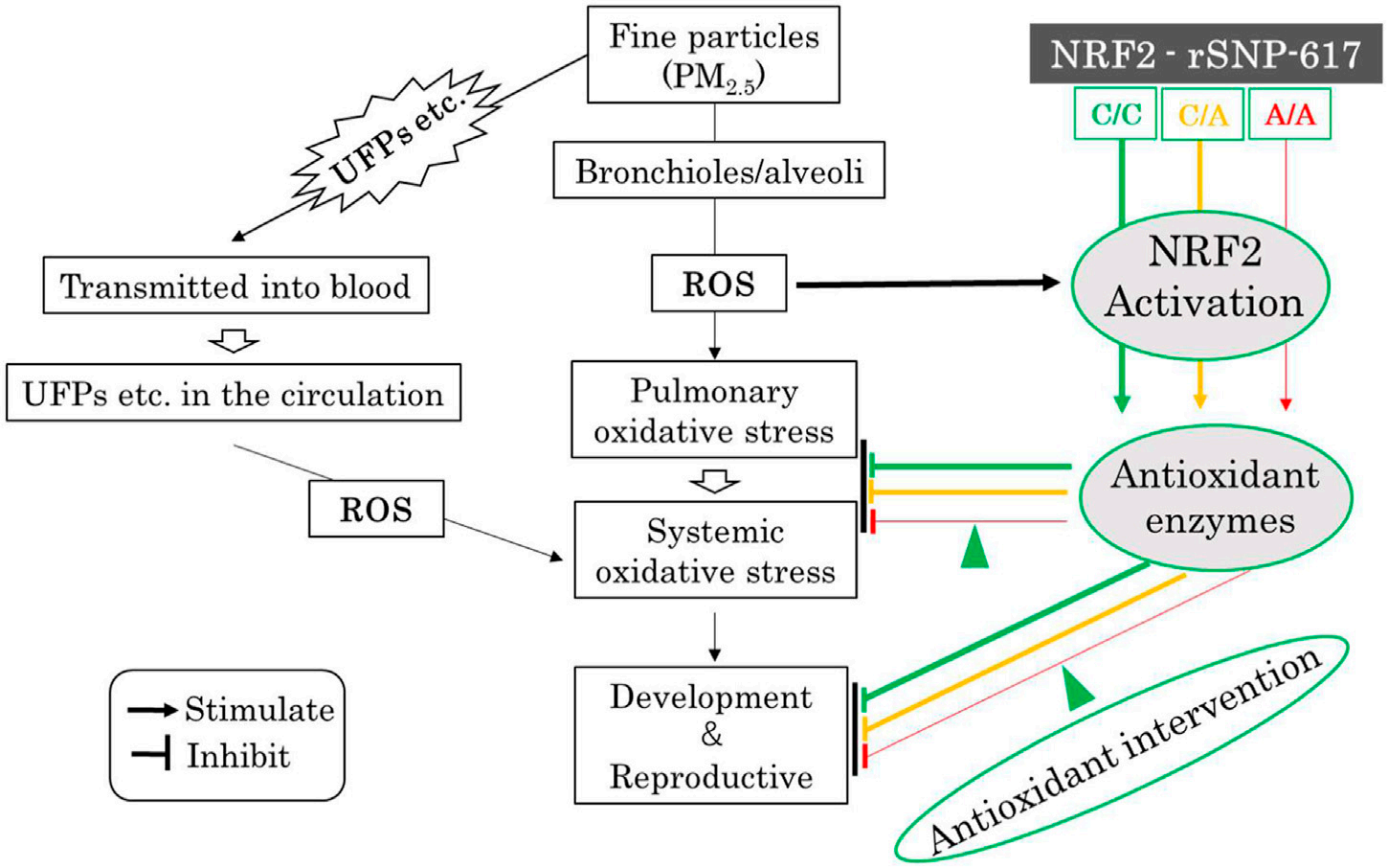
Hypothesis schematic diagram of the potential NRF2 pathways to prevent developmental and reproductive toxicity of fine particles. The colored lines show the putative NRF2 signaling pathway, with the signals being weaker in the order green, orange, and red. ROS: reactive oxygen species, UFPs: ultrafine particles, C/C: wild-type homozygous (c.–617C/C) alleles, C/A: SNP heterozygous (c.–617C/A) alleles, A/A: SNP homozygous (c.–617A/A) alleles; Green arrowhead: antioxidant intervention (NRF2-activating compounds, etc.).

The onset and exacerbation of diseases are regulated by genetic predisposition and environmental factors; some people living in an air-polluted environment are not affected by it and remain healthy. This suggests that there are individual differences in the susceptibility to air pollutants. Many reports mentioned above suggest that *NRF2* polymorphisms may be associated with the development and replication of oxidative stress caused by nanoparticle exposure. It has been reported that the *NRF2* SNP homozygous allele (–617A/A) is a useful biomarker for clinical diagnosis (Okano et al., 2013). In air-polluted areas, it is possible to screen high-risk pregnant women genetically susceptible to oxidative stress using *NRF2* SNP homozygous allele (-617A/A) biomarker; personalized prevention of UFPs-induced oxidative stress at the developmental and reproductive stages, by antioxidant interventions based on NRF2 polymorphisms, may be feasible. Activation of NRF2 represses pro-inflammatory reactions induced by oxidative stress and ameliorates various inflammatory diseases. Many NRF2-activating compounds have been developed or are currently being refined (Suzuki et al., 2020). Figure 2 shows a schematic diagram of the potential of the NRF2 pathway to prevent developmental and reproductive toxicity caused by fine particles.

## Conclusion

This review is a literature review and has not been statistically analyzed. Regarding the health effects of fine particulates, the effects of component analysis have not been analyzed. Although there are these limits, the information summarized in this report highlights the importance of the NRF2-antioxidant pathway and proposes a hypothesis for preventing developmental and reproductive processes implicated in oxidative stress caused by exposure to fine particles in the atmosphere.
